# Energy Preserved Sampling for Compressed Sensing MRI

**DOI:** 10.1155/2014/546814

**Published:** 2014-05-26

**Authors:** Yudong Zhang, Bradley S. Peterson, Genlin Ji, Zhengchao Dong

**Affiliations:** ^1^School of Computer Science and Technology, Nanjing Normal University, Nanjing, Jiangsu 210023, China; ^2^Brain Imaging Laboratory, Department of Psychiatry, Columbia University, New York, NY 10032, USA; ^3^MRI Unit, New York State Psychiatric Institute, New York, NY 10032, USA

## Abstract

The sampling patterns, cost functions, and reconstruction algorithms play important roles in optimizing compressed sensing magnetic resonance imaging (CS-MRI). Simple random sampling patterns did not take into account the energy distribution in *k*-space and resulted in suboptimal reconstruction of MR images. Therefore, a variety of variable density (VD) based samplings patterns had been developed. To further improve it, we propose a novel energy preserving sampling (ePRESS) method. Besides, we improve the cost function by introducing phase correction and region of support matrix, and we propose iterative thresholding algorithm (ITA) to solve the improved cost function. We evaluate the proposed ePRESS sampling method, improved cost function, and ITA reconstruction algorithm by 2D digital phantom and 2D *in vivo* MR brains of healthy volunteers. These assessments demonstrate that the proposed ePRESS method performs better than VD, POWER, and BKO; the improved cost function can achieve better reconstruction quality than conventional cost function; and the ITA is faster than SISTA and is competitive with FISTA in terms of computation time.

## 1. Introduction

Reduction of scan time remains one of the most important areas of emphasis for methodological development in magnetic resonance imaging (MRI) for more than three decades [1, 2]. A variety of fast MRI methods has been developed. One kind of fast MRI techniques reduce scan time by using short repetition time (TR), such as steady state free precession (SSFP) [3], or increasing number of frequency encoding (FE) lines within one TR, such as echo planar imaging (EPI) [4]. These methods acquire all points in *k*-space. Another kind of fast MRI techniques is based on reducing the number of sampling points in *k*-space and includes partial Fourier and parallel MRI [5–7]. Partial Fourier acquires a little more than half of the *k*-space and makes use of the conjugate property of the *k*-space data to fill offline the missing data points by the complex conjugate of the acquired ones [8]. Parallel MRI technique employs an array of surface coils whose spatially varying sensitivities can provide a mechanism of effective spatial encoding that allows one to regularly skip some, for example, every other, phase encoding (PE) lines. Most recently, compressed sensing (CS) was introduced into MRI (CS-MRI) [9], which randomly skips some PE lines, thus reducing scan times.

CS is a technique that acquires signals at sampling rates lower than those indicated by conventional sampling theorems such as the Nyquist theorem, which states that acquisition rates must exceed by 2-fold the highest frequency of the band-limited signal if the signal is to be reconstructed without alias artifacts. When the sampling rate drops below the Nyquist frequency (i.e., when the data are undersampled), alias or folder-over will occur in the high frequency portion of the signal. In many imaging applications such as MRI, in which signals are not band-limited, the acquisition rate of *k*-space data is determined by the desired field of view (FOV). Acquiring data at a rate lower than 1/FOV produces the artifact of image fold-over, portions of the object outside of the FOV will be mapped into the FOV. Yet, acquiring full *k*-space data at a rate that avoids fold-over can be time-consuming. CS allows acquisition of *k*-space signal at random points and lower rates than 1/FOV and reconstruction of the magnetic resonance (MR) images from thus undersampled data [9].

The application of CS to MRI generally entails random undersampling (RAND) of *k*-space data in the PE direction(s), as well as a specialized algorithm for reconstructing the MR images from data that are so drastically undersampled. As RAND will cause noise-like aliasing artifacts, the reconstruction of the CS-MRI is actually a procedure of unaliasing. Previous investigators [9] have proposed a random sampling scheme, named variable density (VD), which samples more densely in the inner than outer region of *k*-space, so that image components with high energy and low frequency alias less than those do with lower energy and higher frequency, thus yielding reconstructed MR images of acceptable quality. Subsequently, several groups have proposed methods to improve the VD sampling patterns so as to optimize reconstruction of CS-MRI [10, 11]. One group [11] based the pattern of RAND on the power spectrum of the *k*-space data of the reference images, showing that it could achieve better image quality than nonoptimized sampling patterns did at the same acceleration rate.

The algorithms for reconstruction of MR images also play important roles in the application of CS to MRI data. The reconstructions of CS-MRI can be obtained by solving constrained optimization problems. The classical approach to such problems is the least squares method, also known as *L*_2_ norm minimization [12], even though this approach may yield poor restoration results for some practical applications, as the unknown coefficients seldom have minimal energy [10]. To enforce the sparsity constraint when solving these underdetermined linear equations, the *L*_1_ norm should be minimized [13]. Special techniques for solving *L*_1_ norm have been developed and include interior points [14], convex set projections [15], message-passing [16], iterative soft thresholding [17], and iterative reweighted least squares [18]. One group proposed specifically for CS-MRI a method that employs nonlinear conjugate gradients and a back-tracking line search [9]. The above mentioned *L*_1_ norm minimization techniques belong to the 1st generation solver. Recently, new CS techniques are proposed. SPGL1 is a Matlab solver for large-scale one-norm regularized least squares [19]. NESTA is a fast and robust first-order method than solves basis-pursuit problems and a large number of extensions (including tv-denoising) [20]. FISTA preserves the computational simplicity of iterative shrinkage/thresholding algorithm (ISTA) but with a global rate of convergence which is proven to be significantly better [21]. SISTA is an extension of ISTA, but it can adaptably choose subband-dependent steps and thresholds [22]. C-SALSA is more general than SPGL1 in the sense that it can be used with any convex regularizer *φ* [23]. FWISTA combines the advantages of generic step method, wavelet-subband-dependent methods, and random shifting technique [24].

The contribution of the present paper is triple: first, we propose a new pattern of data sampling for CS-MRI, which samples the *k*-space data points with statistically high energy based on the reference images. This method is similar to power (Knoll's adapted random sampling pattern method [11]) but prefers to sample high energy points. We name our method energy preserved sampling (ePRESS). Second, we revise the cost function, adding phase correction matrix and region of support (ROS) matrix, to accelerate the convergence and alleviate background noise. Third, to solve the revised cost function, we propose an iterative thresholding algorithm that is as fast as the existing CS-MRI reconstruction algorithms.

## 2. Materials and Methods

### 2.1. Related Work

In Lustig's work [25], they presented the VD function based on a polynomial function of the distance to the origin. Suppose *k*_*y*_ and *k*_*z*_ denote the *k*-space coefficients in the two phase encoding direction and *N* and *M* are the number of phase encoding steps in *y* and *z* direction, the pdf of VD is given by

(1) $$\begin{array}{c} {\left(1-\frac{1}{\sqrt{2NM}}\sqrt{k_{y}^{2}+k_{z}^{2}}\right)}^{p}. \end{array}$$

Here *p* is a parameter to adjust the shape of the PDF. A question arises: how to choose the appropriate *p*? Additionally, suboptimal choosing of *p* leads to significantly low reconstruction quality [11]. Besides, the VD function cannot contain the information of brain structure within a simple mathematical formula. In this study, we developed a nonparametric method that bears the information of reference images, in light of the known fact that reference image and test image are similar; we can design the sampling trajectory from the reference images which is proven to perform better than existing sampling methods.

### 2.2. ePRESS Sampling

The magnitudes of the *k*-space data points can be regarded as the power distribution of the *k*-space signals. The ePRESS is based on the hypotheses that the *k*-space data are correlated to the structure of the object, in addition to the parameters of the pulse sequences, and that similar structures of the objects will result in statistically similar *k*-space data. ePRESS sampling patterns were obtained based on probability distribution function (PDF) in three steps.

**Figure 1:**
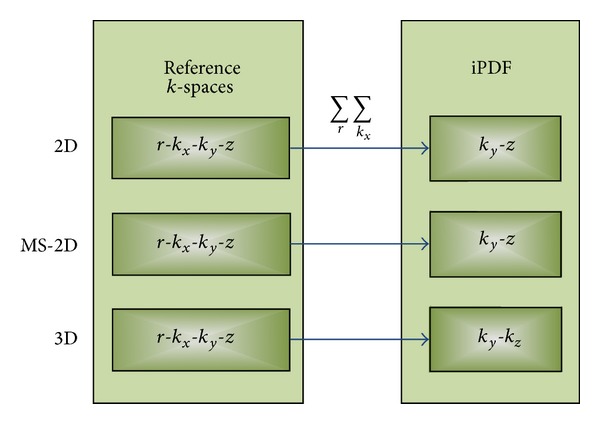
From references *k*-spaces to iPDF (*x*, *y*, *z*: the 3D coordinates in image space; *k*_*x*_, *k*_*y*_, *k*_*z*_: the 3D coordinates in *k*-space; *r*: the reference index).

*Step 1 (determine the initial probability distribution function (iPDF)).* We generated the iPDF by summarizing the magnitudes of *k*-space data of the reference scans along the readout direction (*k*_*x*_) and by summarizing all the references, before normalizing to unity.  Figure 1 shows how to form an iPDF from reference *k*-spaces.

*Step 2 (calculate the windowed probability distribution function (wPDF))*. The iPDF tends to emphasize the high-energy and low-frequency data points at the center of *k*-space and ignore high-frequency data points at the outer portions of *k*-space, which risks a loss of high frequency information in the reconstructed images. We addressed this problem by applying a hamming window function to the iPDF (3), a function that is optimized to minimize the maximum sidelobe. The 2D hamming window function is given as

(2) ω(m,n)=(0.54−0.46cos⁡(2πmN))×(0.54−0.46cos⁡(2πnN)), 0≤m,  n≤N.

**Figure 2:**
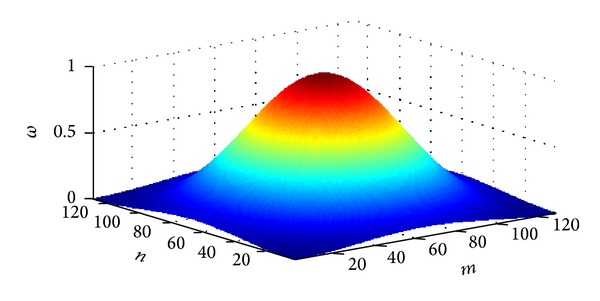
A 128 × 128 hamming window.

Here *m*, *n* are auxiliary parameters of the window and *ω* is the window function. The advantages of hamming windows (Figure 2) are that they can minimize the maximum sidelobe.

We then calculated the wPDF as

(3) $$\begin{array}{c} \text{wPDF}=\frac{\text{PDF}}{\omega {\left(m,n\right)}^{\alpha }}, \end{array}$$

where *α* is an empirical parameter adjusting the distribution of the wPDF, in which values of *α* larger than 1 increase the probability of sampling the outer portion of *k*-space. The hamming window gives wPDF incoherence that is the required component of CS.

**Figure 3:**
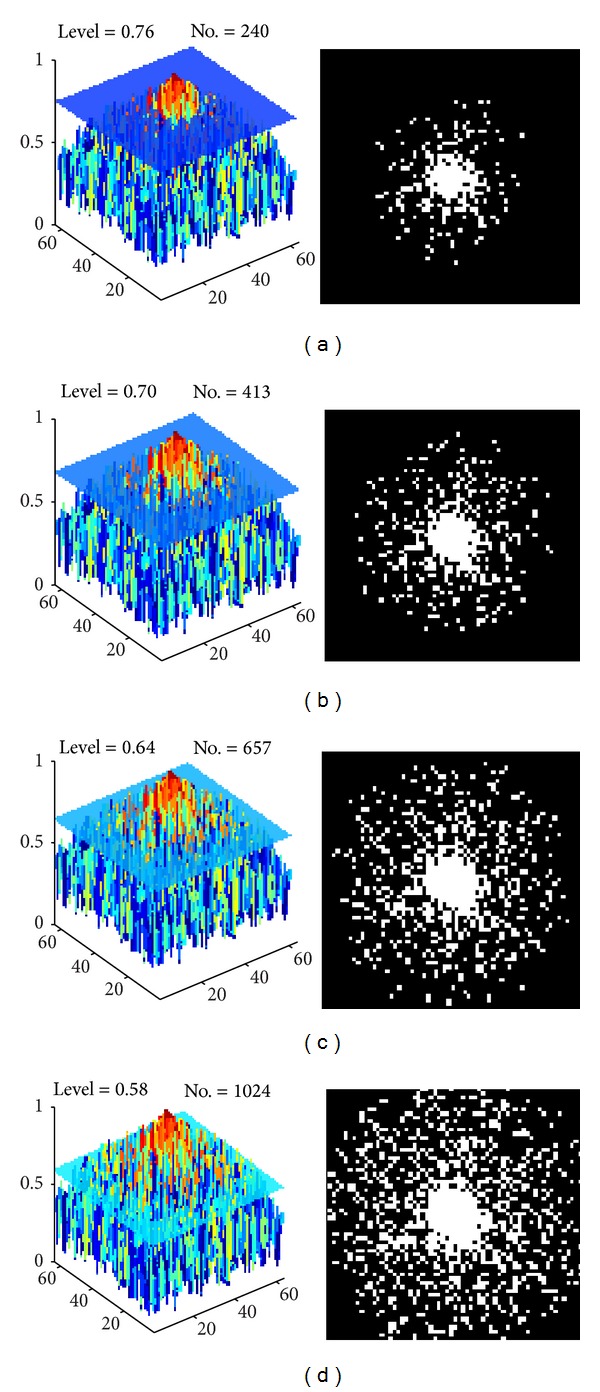
From wPDF to sampling patterns at 4 different levels: (a) 0.76, (b) 0.70, (c) 0.64, and (d) 0.58.

*Step 3 (select the indexes of the sampling points).* Indexes of sample points were selected from the wPDF according to a level or threshold determined by the number of data points (Figure 3). The points above the threshold represent high-energy points that will be selected. The threshold decreases until predetermined number of sampling points are reached. There is no exact formula to describe the curve of the number sampled against the threshold. The users need to employ 1D numerical optimization method (such as Newton-Raphson method or BFGS method) to get the exact threshold for a given acceleration factor.

**Figure 4:**
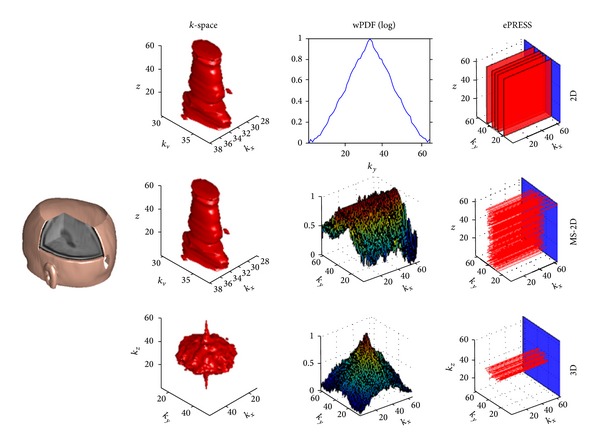
The sampling designs of 2D, MS-2D, and 3D MRI.

The ePRESS sampling method can be applied to 2D, MS-2D, and 3D MRI. For 2D MRI, we perform 1D undersampling in the same way on each slice. For MS-2D, we performed different 1D undersampling on each slice. 3D MRI is the preferred imaging method because (1) it is more flexible to choose random points, (2) the *k*_*x*_-*k*_*y*_-*k*_*z*_ plane is sparser, and (3) the *k*_*x*_-*k*_*y*_-*k*_*z*_ plane resists in-plane motion efficiently; however, it is still sensitive to out-of-plane rotation.  Figure 4 shows the whole procedure of 2D, MS-2D, and 3D sampling. In the following text, we take one slice from 3D MRI as example for simplicity and suppose *k*_*y*_ and *k*_*z*_ are PE directions. This hypothesis can reach the fullest expression as found in other literature [9, 11, 25].

### 2.3. Improved Cost Function

Mathematically, images are reconstructed by solving a constrained optimization problem [9, 26]. The cost function (CF) is written as

(4) min⁡ ||Ψx||1s.t.  ||Fux−y||2<ε,

where *ψ* denotes the sparse transform matrix, *F*_*u*_ denotes the undersampled Fourier transform, *x* denotes the estimated signal or image, *y* denotes the measured full *k*-space data from the MRI scanner, *ε* controls the fidelity of reconstruction to the measured data, and s.t. denotes “subject to.”

We improved above cost function by employing ROS and the phase correction matrix [27, 28] in order to accelerate convergence of the estimation and to reduce background noise [29]. The phase correction matrix is obtained similar to the PCCS method, using symmetric points near central rows of *k*-space. Suppose *K*_*s*_ represents the symmetric points of the *k*-space and *I*_*s*_ represents its corresponding image obtained by inverse discrete Fourier transform (IDFT). The phase correction matrix can be written as

(5) $$\begin{array}{c} I_{s}=\text{IDFT}\left(K_{s}\right),\\ P=\exp\left(-i*\text{angle}\left(I_{s}\right)\right). \end{array}$$

The improved cost function (ICF) is therefore given as

(6) min⁡ ||Ψx||1s.t.  {||FuPx−y||2<ε,x=Sx,

where *P* is a phase correction matrix whose entries give the estimated phase of each pixel [30] and *S* is a matrix defined as element 1 corresponding to the ROS. *S* can be obtained from a previous coarse scan or other imaging modality. The equation *x* = *Sx* defines the region containing nonzero signal intensities.

### 2.4. Reconstruction of CS-MRI

Conventional methods such as FISTA and SISTA are not applicable for the improved cost function. We proposed an iterative threshold algorithm (ITA) to solve formula (6) by iteratively finding successive approximations to the solution.

**Step 1 (initialization)**

Suppose *Max*⁡_ITER_ denotes the maximum number of iterations, *T* denotes a series of numerical thresholds decreasing linearly, and *T*_*i*_ and *T*_*f*_ represent the initial and final value of *T*, respectively. Every component of *T* can be written as

(7) T(k)=Ti−Ti−TfMaxITER−1×(k−1),k=1,2,…,MaxITER.

**Step 2 (set *x*_0_)**

The initial point *x*_0_ is the IDFT of the zero-padding of *y*.

**Step 3 (minimize 1-norm of *ψ*(*x*))**

Transform *x* to the sparse domain; keep the sparsity coefficients unchanged if they are greater than the *i*th threshold *T*_*i*_; otherwise, set them to zero:

(8) $$\begin{array}{c} x\left(k+1\right)=\psi^{-1}\left[\psi \left(x\left(k\right)\right)>T_{i}\right]. \end{array}$$

Here *x*(*k*) represents the value of *x* at *k*th step.

**Step 4 (satisfy the 1st subjection)**

Transform *x* to the *k*-space; replace the undersampled position with the realistic sampling results *y* and transform back to the image space:

(9) $$\begin{array}{c} x\left(k+1\right)=P^{-1}F^{-1}\left[FP{\left(x\left(k+1\right)\right)}_{u}\times y\right]. \end{array}$$

**Step 5**

Satisfy the 2nd subjection:

(10) $$\begin{array}{c} x\left(k+1\right)=S\times x\left(k+1\right). \end{array}$$

**Step 6**

Repeat until termination criteria are reached.

This method is simple and easy to realize. Almost every CS-MRI method uses discrete Fourier transform (DFT) and discrete wavelet transform (DWT) which are fast and save memory storage; however, some methods have to use other time-consuming techniques which cost lots of computation resources. Our ITA method only uses the two mature techniques (DFT and DWT) and can achieve the reconstruction goal, so it is reasonable to expect it as comparable to existing fastest CS-MRI algorithms.

The proposed ITA method is different from and cannot be replaced with ISTA and its derivations. First, ITA is only suitable for the proposed improved cost function, which ISTAs cannot solve. Second, ISTA combined the Fourier encoding matrix and wavelet transform matrix together, while our ITA method treated them in different steps. Third, ISTA has a parameter *L* that should be greater or equal to the Lipschitz constant of the gradients of ||*F*Ψ*x*||_2_^2^, and the proposed ITA method has two parameters *T*_*i*_ and *T*_*f*_.

The termination criteria of ITA are set as follows: when the *x* is no longer improved (measured by a fixed amount *F*) over continuous *I* iterations or the iteration reaches the predefined maximal iterative number, then the algorithm should stop:

(11) if  ||xk+1−xk||≤F  over  I  continuous  iterations  or  k>MaxITER,then  algorithm  stops.

This type of termination criterion is commonly used in other literature [31–33].

### 2.5. Implementation and Evaluation

We implemented all experiments in Matlab 2013a on a 64bit core i3 laptop, 2 GHz clock rate, 2 GB random-access memory, and Win 7 OS. The method was tested and demonstrated using 2D digital phantoms and 2D* in vivo* MRIs from the brains of healthy participants. The MRI protocol was approved by the New York State Psychiatric Institute IRB (Institutional Review Broad) and written informed consent was obtained from the subjects prior to MR scans.

**Figure 5:**
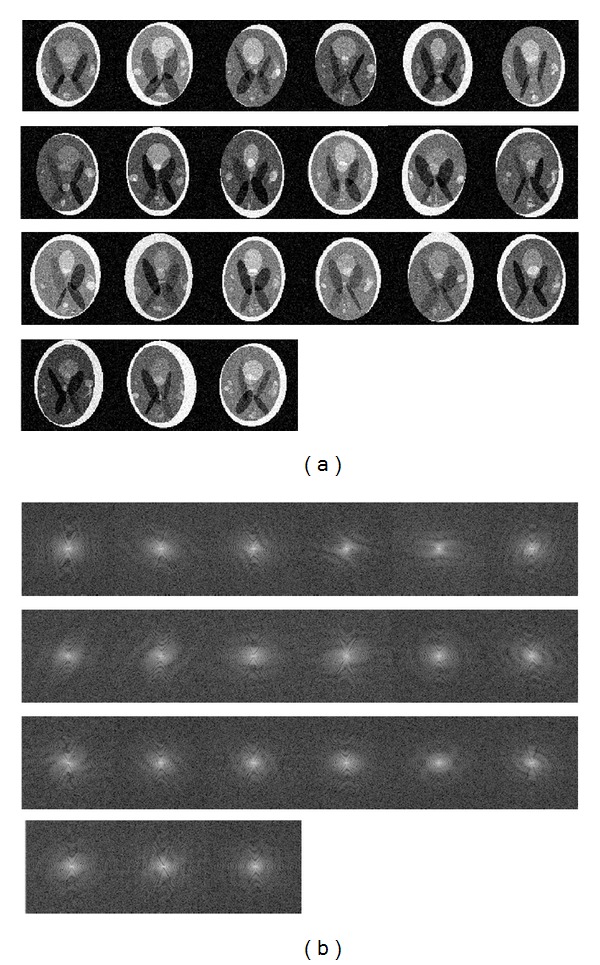
Materials of 2D SL Phantom CS-MRI: (a) twenty reference phantom images and a test phantom image and (b) their corresponding *k*-spaces.

In the first experiment, we conducted a 2D phantom experiment to show each step of our proposed method. The sparse transform is chosen as haar wavelet with 3 decomposition levels [34, 35]. First, we generated 21 different 128 × 128 images from the Shepp Logan head phantom using image distortion, translation, and rotation operators randomly. The six important parameters of the ellipse are *A* (additive intensity value of the ellipse), *a* (the length of horizontal semiaxis of the ellipse), *b* (the length of vertical semiaxis of the ellipse), *x*_0_ (the *x*-coordinate of the center of the ellipse), *y*_0_ (the *y*-coordinate of the center of the ellipse), and *φ* (the angle between the horizontal semiaxis of the ellipse and the *x*-axis of the image). We added Gaussian white noise with mean 0 and variances vector as $\left[\begin{array}{cccccc} 0.1 & 0.05 & 0.05 & 0.05 & 0.05 & 10 \end{array}\right]$ to them. Twenty images were used as reference images and the last as the test image (Figure 5). To assess the quality of different sampling methods, we calculated the correlation coefficient between the *k*-space of test image and the estimated map (obtained using both VD and ePRESS). Afterwards, we applied VD, power, and ePRESS samplings, each with the corresponding sampling ratio (reciprocal of acceleration factor) ranging from 0.5 to 0.05 in −0.05 decrements (Table 1). We calculated the energy preserving ratio (EPR, defined as the energy ratio of the sampled *k*-space points to the full *k*-space) against acceleration factors for each of the three sampling patterns. The parameter *α* in ePRESS is set as 0.8.

The goal of the second experiment is to assess performance of different sampling methods using 2D* in vivo* brain images from healthy participants. We used conventional CF and FISTA reconstruction algorithm. First we used eleven 256 × 256 transverse MRI brain images, 10 of which were used as reference to generate the ePRESS (*α* = 1.4) pattern. The last was used as a test image, which was transformed to *k*-space and then resampled with acceleration factors of 4 to provide three different sampling patterns, namely, low resolution (scan the central part of *k*-space), VD, and ePRESS. Next, we used eleven 256 × 256 sagittal brain images with the same setting as aforementioned and compared ePRESS with BKO (Seeger's Bayesian *k*-space optimization) [10] and power methods [11]. The sparse transform is chosen as bior4.4 wavelet at level 6 [36–38]. Two popular error indicators, median absolute error (MAE) and median square error (MSE), were used for the assessment.

The third experiment is designed with the aim of comparing conventional cost function with the proposed ICF. For conventional CF, we solved it by FISTA. For the proposed ICF, we solved it by the proposed ITA method, since FISTA did not involve phase correction matrix and ROS matrix. We used VD and ePRESS sampling methods. The sparse transform is chosen as bior4.4 wavelet at level 6.

Finally, we compared ITA method with FISTA and SISTA in terms of computation time. ITA is designed specifically to solve the improved cost function as formula (6). FISTA and SISTA can only solve the conventional CF as formula (4). We iteratively performed all algorithms and recorded the consumed time under the condition when each algorithm obtained nearly the same MAE.

## 3. Results

### 3.1. 2D SL Phantom Illustration

**Figure 6:**
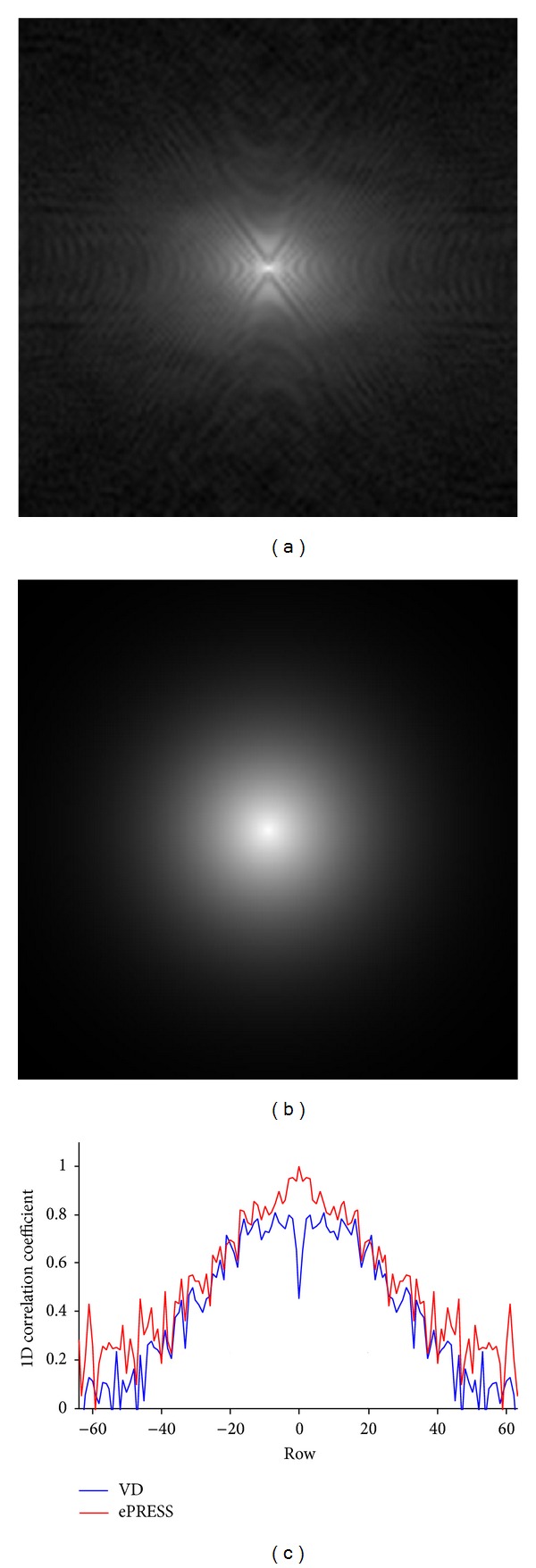
Comparisons of ePRESS and VD: (a) ePRESS map, (b) VD Map, and (c) 1D row-wise coefficients of correlation with the *k*-space data of test phantom.

Figure 5 shows the 20 reference images and 1 test image. They differ considerably not only in the spatial image but also in the *k*-spaces. Figures 6(a)-6(b) show the differences between ePRESS iPDF and VD PDF. Parameter *p* of VD is chosen as 10 by trial-and-error method.  Figure 6(c) shows the 1D row-wise correlation coefficients between the maps (ePRESS iPDF and VD PDF) and *k*-space of the test image. We found that ePRESS is more highly related to the test image than VD. The 2D correlation coefficient is 0.942 for ePRESS, higher than that for VD (0.531).

It is easily perceived that in Figure 6(a) there are regular textures that correspond to the brain structure in the image space, which demonstrate that the optimal PDF maps found by ePRESS are much more complicated than VD. The correlation coefficient curve in Figure 6(c) indicates that the ePRESS map is more similar to the *k*-space data of the test phantom than VD, and therefore the reference images can act as* a priori* information that predicts the *k*-space of the individual brain to be scanned and guides the subsequent design of sampling points.

**Figure 7:**
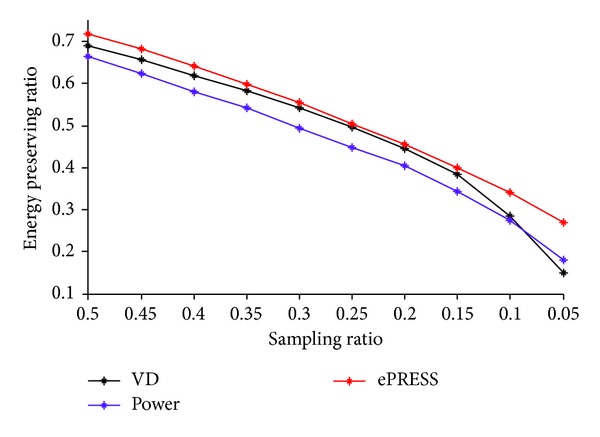
The curve of EPR versus sampling ratio.

Then, we generate the sampling trajectory from ePRESS iPDF, VD PDF, and power PDF. Table 1 shows the sampling points are sparser as the sampling ratio decreases.  Figure 7 shows the curve of EPR versus the sampling ratio. It proves the energy of sampling points of ePRESS is always higher than VD and power methods, despite the fact that the latter two methods provide denser sampling trajectories than ePRESS does.

Results in Table 1 indicate that power is similar to ePRESS, but the main difference falls within the procedures from PDF/iPDF map to sampling points. Power used a random number generator, and a point was selected if the random number was lower than the corresponding value of PDF map. The proposed ePRESS first used hamming window to change the iPDF map to wPDF map so that the high frequency points stand more chances to be selected and then used a thresholding method to select the sampling points.

Figure 7 indicates that the ePRESS method is superior to the other two methods in the respect of preserving energy, especially when the sampling ratio is less than 0.15. Besides, the EPR curve of ePRESS decreases dramatically if the sampling ratio is less than 0.1. Therefore, the appropriate acceleration factor of the proposed ePRESS method is less than 10.

### 3.2. Sampling Trajectories Comparison

**Figure 8:**
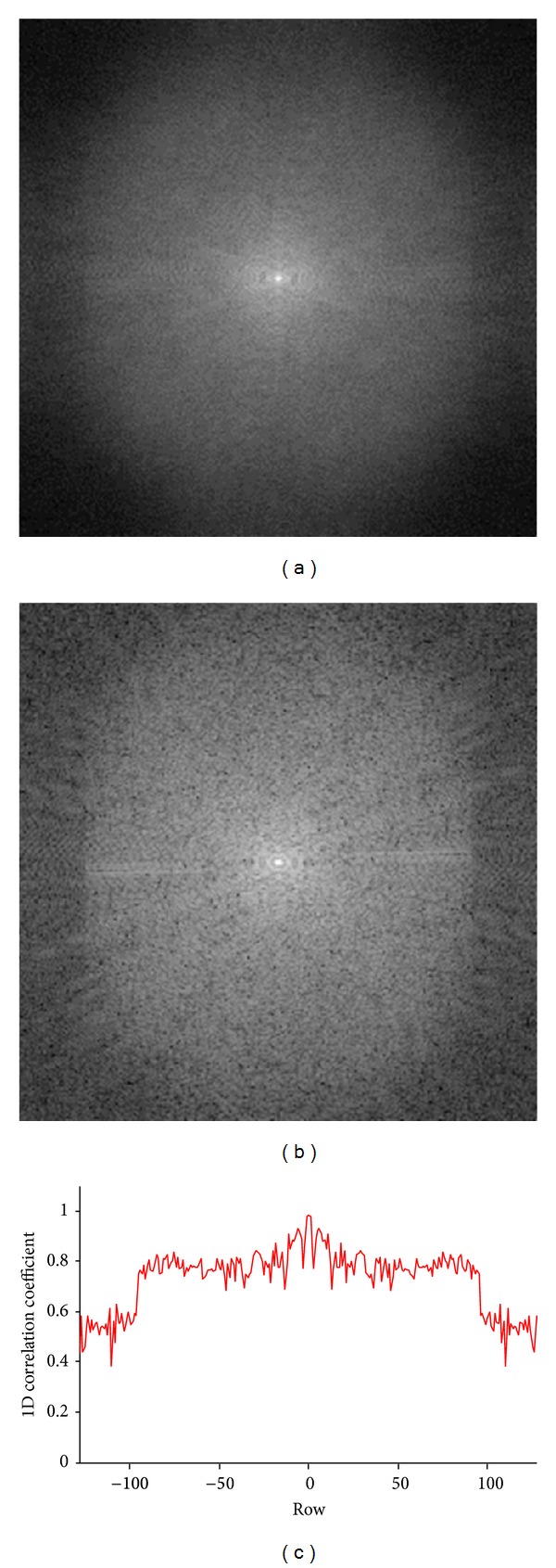
Preprocessing of ePRESS: (a) ePRESS iPDF; (b) *k*-space of test image; (c) row-wise coefficients correlation between (a) and (b).

Figure 8 shows the preprocessing of ePRESS.  Figure 8(a) gives the iPDF obtained from 10 reference images.  Figure 8(b) gives the *k*-space of the test image that looks similar to the iPDF, which demonstrates the effectiveness of ePRESS.  Figure 8(c) shows the 1D row-wise correlation coefficients curve between the ePRESS iPDF and the *k*-space of test image. Their 2D correlation coefficient is 0.949.

**Figure 9:**
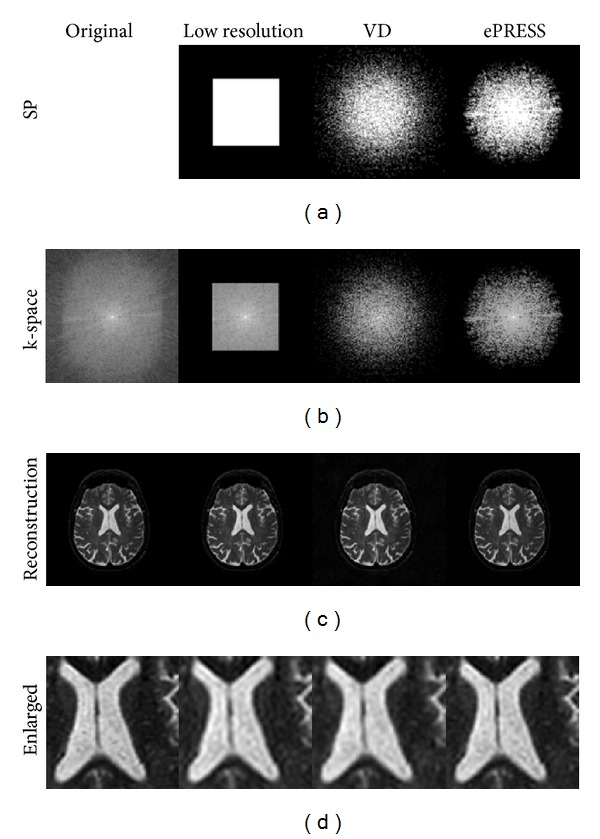
Sampling method comparison on transverse plane with 4x acceleration: the sampling patterns (a), *k*-space (b), reconstruction (c), and the zoom-in of the reconstruction (d).

Three different sampling patterns: low resolution, VD, and ePRESS, with their corresponding *k*-spaces and CS reconstructions shown in Figure 9. Conventional CF and FISTA were employed. The results showed that low resolution lost high frequency information of the original image, resulting in edge blurring. VD got clear brain with noticeable background noise. Nevertheless, the proposed ePRESS method reconstructed the clearest brain.  Table 2 shows the MAE and MSE between the reconstruction and ground-truth image. The data indicate that ePRESS obtains the best recovery quality.

**Figure 10:**
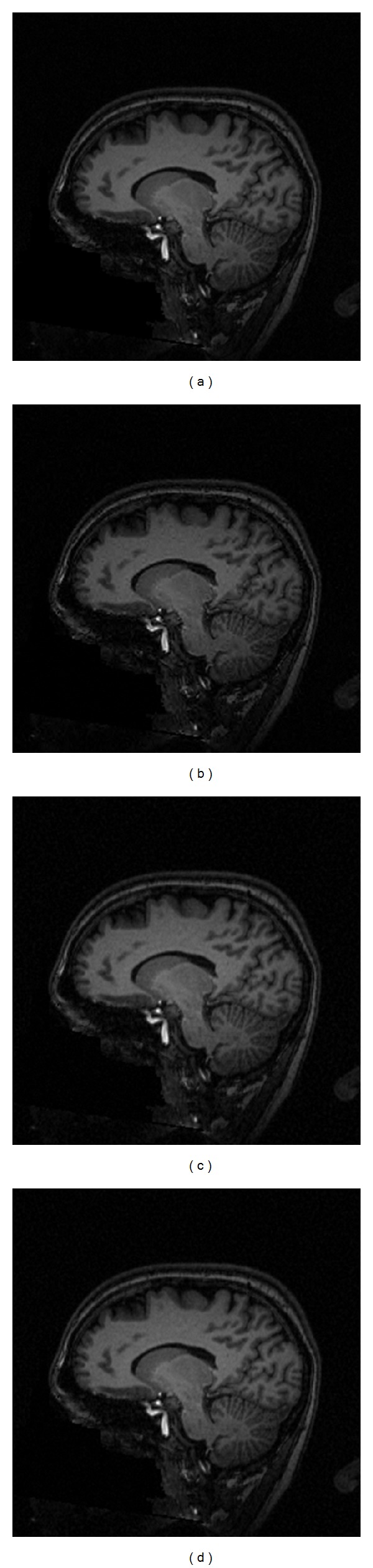
Sampling comparison on sagittal plane of 2D normal brain: (a) original; (b): BKO (EPR = 0.68, MAE = 2.84, and MSE = 16.98); (c) power (EPR = 0.69, MAE = 2.60, and MSE = 12.18); (d) ePRESS (EPR = 0.72, MAE = 2.29, and MSE = 9.24).

Except above conventional methods, we compared ePRESS with BKO [10] and power methods [11] on a sagittal section of a normal brain (Figure 10). Eleven sagittal planes are selected, ten of which as reference images and the rest as test image. Parameters are set in the same way as above. Our ePRESS method achieved the highest EPR as 0.72 and the least MAE as 2.29 and MSE as 9.24.

### 3.3. Cost Function Comparison

**Figure 11:**
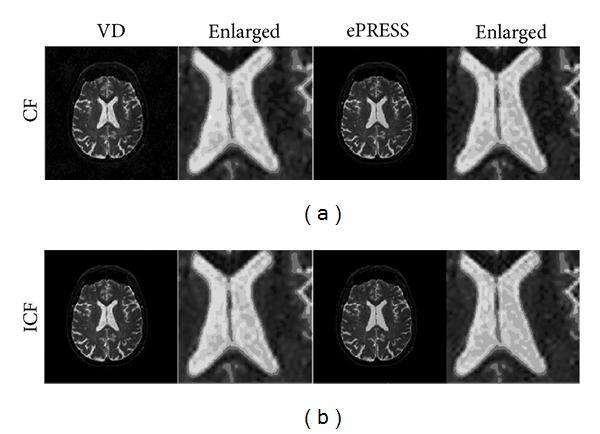
Comparison between conventional CF and ICF with 4x acceleration.

Figure 11 and Table 3 illustrate comparison between CF and ICF. They show that the proposed ICF model achieves better reconstruction than conventional CF does. Besides, “ePRESS + ICF” model achieved better result results than “ePRESS + CF,” “VD + CF,” and “VD + ICF.”

Revisiting what has been mentioned in Figure 9, Figure 11 again indicates that the proposed ePRESS method outperforms VD due to the ignorable noises and well-preserved edges as shown in the gap of the lateral ventricles located in the center of the brain.

### 3.4. Reconstruction Algorithm Comparison

Table 4 shows the comparison of ITA with FISTA and SISTA. ITA is employed to solve the ICF as formula (6), while FISTA and SISTA are employed to solve conventional CF as formula (4). As the iteration runs till the MAE between the reconstruction and ground-truth image reached predefined value (2.3, 2.2, and 2.1), the time of FISTA, SISTA, and ITA are recorded. We found that SISTA converges the most slowly due to its subband processing property. FISTA converges the fastest. However, those two methods cannot be applied to solve our model. The ITA is comparable to FISTA and much faster than SISTA.

## 4. Discussion

The ePRESS method was motivated by the idea of energy-preserving. The high-value energy points in the central part of *k*-space correspond to the common features and the low-value energy points to individual features. The points corresponding to common features increase remarkably faster than those points to individual features during which *k*-space data of the references are summed up. Therefore, the iPDF can be regarded as a map of common features and the ePRESS can retain high signal-to-noise ratio (SNR) of the reconstructed CS-MRI because high energy points are preserved. The individual features of the brains seemingly do not benefit as much as the common features from the ePRESS from the point of view of the PDF, as preference of data selection was given to high energy points. But information of the individual features is duly included in the selected points because of the property of Fourier encoding or the relation between the image and the *k*-space data. This means that ePRESS does not introduce extra loss of information for the individual features.

The above analysis and conclusion are of importance for the applications of ePRESS. The reference scans used for the PDF were from healthy subjects and the ePRESS tends to preserve energy contributed from global or common features of the brains. Therefore, the ePRESS method is most suitable for MRI studies of normal appearing brains such as those in psychiatric studies. In most cases of occupying lesions, common features of the brains are still dominant and the ePRESS method is expected to perform well-preserving energy and retaining SNR. The advantage of ePRESS may be slightly compromised, however, when the structure of the brain severely deviates from normal.

A concern about the ePRESS is whether the size of a head can affect the performance of the ePRESS. Indeed, the energy distributions of *k*-space data will shrink or expand when the sizes of the brains increase or decrease, according to the property of Fourier transform. However, our method used reference images of heads of different sizes, and the common features of these energy distributions will be preserved in the sense of statistics. Therefore, small changes in the head size will not influence the performance of ePRESS. This was demonstrated by the simulation using Shepp-Logan digital phantom.

A shortcoming of the ePRESS is that, in some extreme cases (sampling ratio < 0.1), the number of selected high frequency points will decrease sharply, and, consequently, the recovery of high-frequency is not ensured. We note that the 10x acceleration is not practical in most real cases. Should there be such a need, the factor *α* in the wPDF should be increased to sample more high frequency points.

The number of reference *k*-space datasets is limited by the size of available images with the same parameters of pulse sequence. The more reference datasets are used, the more accurate the PDF map will be. There is no need to adjust the reference images to the same contrast and position, according to the shift invariability of DFT.

Our scheme is suitable for 3D scan, in which the random points are located in the PE plane not the FE direction. Besides, the proposed method can be revised and used for 2D scan where only 1 direction is set as PE and the other direction is set as FE.

Parameter *α* is an empirical parameter adjusting the distribution of the wPDF. For the 2D phantom, which contains mostly the piecewise-constant objects, we found *α* = 0.8 is appropriate. For the 2D realistic brain, which contains complex textures, we found *α* = 1.4 is suitable. Besides the content of the imaging subject, the acceleration factor also influenced the selection of *α*. In practice, we used trial-and-error method to choose the optimal value of *α*; namely, let *α* vary from the lower bound 0.5 to upper bound 2 with increment as 0.1 and choose the one corresponding to the best reconstruction quality. How to determine the best value remains a challenge for the proposed algorithm, but we will try to solve it in our future research.

The contribution of the paper falls within the following three points: (1) we designed a new variable sampling method, ePRESS, based on the energy-preserving concept; (2) we introduced an improved cost function model as formula (6) that took into account the phase correction matrix and ROS; and (3) we proposed an ITA method to solve our ICF model. In the experiments, we compared them with existing methods and models using data sets from 2D phantom and* in vivo* 2D MR images.

The future research contains the following issues: (1) using more patients to validate our method; (2) generalizing our method to other MR modalities including fMRI, DTI, and MRSI; (3) comparing haar and bior4.4 with other wavelets and making a tentative test to include the complex dualtree wavelet for potential better results; (4) design an automatic method to choose the optimal decomposition level.

## Figures and Tables

**Table 1:** The sampling points of VD, power, and ePRESS.

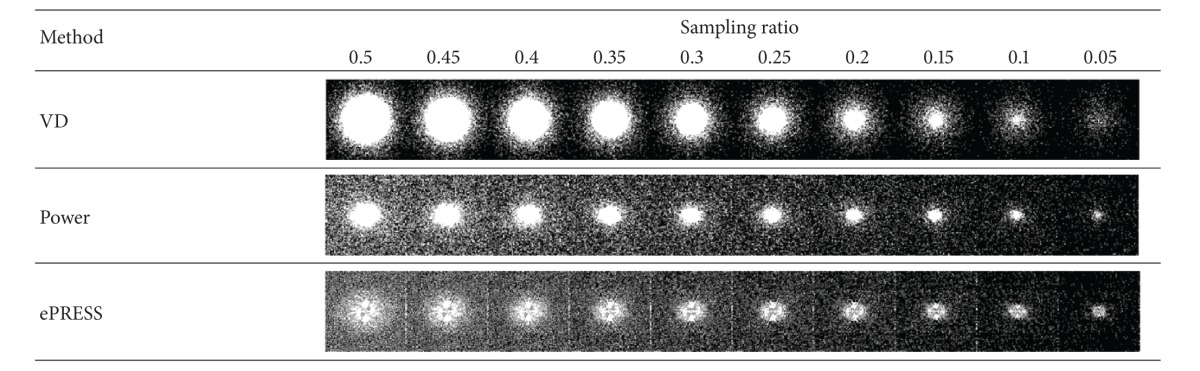

**Table 2:** Sampling method comparison on transverse planes of 2D normal brains with 4x acceleration.

Sampling methods	MAE	MSE
Low resolution	2.56	26.02
VD	2.70	14.92
ePRESS	**2.05**	**11.14**

**Table 3:** Comparison between conventional CF and ICF with 4x acceleration.

Sampling	Cost function	MAE	MSE
VD	Conventional CF	2.70	14.92
	ICF	**2.65**	**14.30**

ePRESS	Conventional CF	2.05	11.14
	ICF	**1.99**	**10.72**

**Table 4:** Computation time comparison on 2D normal brain with 4x acceleration.

MAE	Time (s)		
	FISTA	SISTA	ITA
2.3	6.3	41	9.1
2.2	8.8	75	11
2.1	14	137	19

## Acronyms

(F)(S)ISTA: (Fast) (subband adaptive) ISTA
(I)CF: (Improved) cost function
(I)DFT: (Inverse) discrete Fourier transform
CS: Compressed sensing
DWT: Discrete wavelet transform
EPI: Echo planar imaging
EPR: Energy preserving ratio
ePRESS: Energy preserving sampling
FE: Frequency encoding
FOV: Field of view
ISTA: Iterative shrinkage/thresholding algorithm
ITA: Iterative thresholding algorithm
MAE: Median absolute error
MRI: Magnetic resonance imaging
MS: Multislice
MSE: Median square error
PE: Phase encoding
RAND: Random sampling
ROS: Region of support
SSFP: Steady state free precession
TR: Repetition time
VD: Variable density.

## Highlights

(1) We propose ePRESS sampling method based on energy-preserving concept; (2) we improve the cost function by introducing phase correction and region of support matrix, to accelerate the convergence and alleviate background noise; (3) to solve the improved cost function, we propose ITA method that is comparable to FISTA.
